# A Novel *ATP6V0A2* Mutation Causing Recessive Cutis Laxa with Unusual Manifestations of Bleeding Diathesis and Defective Wound Healing

**DOI:** 10.4274/tjh.galenos.2018.2018.0325

**Published:** 2019-02-07

**Authors:** İlker Karacan, Reyhan Diz Küçükkaya, Fatma Nur Karakuş, Seyhun Solakoğlu, Aslıhan Tolun, Veysel Sabri Hançer, Eda Tahir Turanlı

**Affiliations:** 1 İstanbul Technical University, Graduate School of Science, Engineering and Technology, Department of Molecular Biology-Genetics and Biotechnology, İstanbul, Turkey; 2 İstanbul Medeniyet University, Department of Molecular Biology and Genetics, İstanbul, Turkey; 3 İstanbul Bilim University, Faculty of Medicine, Department of Hematology, İstanbul, Turkey; 4 İstanbul University, İstanbul Faculty of Medicine, Department of Histology and Embryology, İstanbul, Turkey; 5 Boğaziçi University, Department of Molecular Biology and Genetics, İstanbul, Turkey; 6 İstanbul Bilim University, Department of Molecular Biology and Genetics, İstanbul, Turkey; 7 İstanbul Technical University, Department of Molecular Biology and Genetics, İstanbul, Turkey

**Keywords:** ATP6V0A2, Cutis laxa, Wound healing, Bleeding diathesis, Whole exome sequencing

## Abstract

**Objective:**

Autosomal recessive cutis laxa type IIA (ARCL2A) is a rare congenital disorder characterized by loose and elastic skin, growth and developmental delay, and skeletal anomalies. It is caused by biallelic mutations in *ATP6V0A2*. Those mutations lead to increased pH in secretory vesicles and thereby to impaired glycosyltransferase activity and organelle trafficking. We aimed to identify the genetic and molecular cause of the unexpected hematological findings in a Turkish family.

**Materials and Methods:**

We performed clinical, genetic, and histological analyses of a consanguineous family afflicted with wrinkled and loose skin, microcephaly, intellectual disability, cleft lip and palate, downslanting palpebral fissures, ectopia lentis, bleeding diathesis, and defective wound healing.

**Results:**

Linkage analysis using SNP genotype data yielded a maximal multipoint logarithm of odds score of 2.59 at 12q24.21-24.32. Exome sequence analysis for the proband led to the identification of novel homozygous frameshift c.2085_2088del (p.(Ser695Argfs*12)) in *ATP6V0A2*, within the linked region, in the two affected siblings.

**Conclusion:**

Our patients do not have gross structural brain defects besides microcephaly, strabismus, myopia, and growth or developmental delay. Large platelets were observed in the patients and unusual electron-dense intracytoplasmic inclusions in fibroblasts and epidermal basal cells were observed in both affected and unaffected family members. The patients do not have any genetic defect in the *VW*F gene but von Willebrand factor activity to antigen ratios were low. Clinical findings of bleeding diathesis and defective wound healing have not been reported in ARCL2A and hence our findings expand the phenotypic spectrum of the disease.

## Abstract (tr)

**Amaç:**

Otozomal çekinik kutis laksa tip IIA (ARCL2A) nadir görülen ve doğuştan bir hastalık olup, gevşek ve elastik deri, büyüme ve gelişme geriliği, iskelet anomalileri ile karakterizedir. *ATP6V0A*2 genindeki biallelik mutasyonlar hastalığa sebep olmaktadır. Bu mutasyonlar salgı veziküllerinde pH artışına yol açtığından, glikoziltransferaz aktivitesi ve organel trafiği bozulur. Bu çalışmada beklenmedik hematolojik bulguları da olan Türk ailede hastalığın genetik ve moleküler sebeplerinin bulunması amaçlanmıştır.

**Gereç ve Yöntemler:**

Akraba ebeveynin iki çocuğunda buruşuk ve gevşek deri, mikrosefali, zihinsel yetersizlik, yarık dudak ve damak, aşağı eğimli palpebral fissür, mercek dislokasyonu, kanama eğilimi ve yetersiz yara iyileşmesi bulguları görüldü ve klinik, genetik ve histolojik analizler gerçekleştirildi.

**Bulgular:**

SNP genotip verisi kullanılarak yapılan bağlantı analizi sonucunda 12q24.21-24.32 kromozomal bölgesinde en yüksek çok noktalı LOD skor olan 2,59 elde edilmiştir. Bu bölgede indeks hastanın ekzom verilerinde, her iki hastada da homozigot durumda olan ve daha önce bildirilmemiş *ATP6V0A2* geninde çerçeve kaymasına yol açan c.2085_2088del (p.(Ser695Argfs*12)) varyantı tespit edildi.

**Sonuç:**

Hastalarda mikrosefali dışında yapısal beyin bozukluğu, şaşılık, miyop, büyüme veya gelişme geriliği yoktur. Yalnızca hastalarda büyük hacimli trombositler, hem hasta hem de sağlıklı bireylerde ise fibroblast ve epidermal bazal hücrelerde alışılmadık elektron-yoğun intrasitoplazmik inklüzyonlar görülmüştür. Hastalarda *VWF* gen bozukluğu görülmemiş olup, von Willebrand faktörü aktivite/antijen oranı düşüktür. Kanama eğilimi ve yetersiz yara iyileşmesi daha önce ARCL2A hastalığında rapor edilmemiş olup, bulgularımız hastalığın fenotipik spektrumunu genişletmektedir.

## Introduction

Cutis laxa (CL) is a group of connective tissue abnormalities characterized by sagging, inelastic, and wrinkled skin [1]. These skin anomalies result from structural defects of elastic fibers. Congenital CL can be inherited as either an X-linked recessive, autosomal dominant or autosomal recessive trait. Autosomal recessive CL is the most severe type of the disease and displays the highest clinical and genetic heterogeneity. To date, causative mutations in at least eight genes have been identified for autosomal recessive CL: *ALDH18A1, ATP6V0A2, ATP6V1A, ATP6V1E1, FBLN4, FBLN5, LTBP4, *and* PYCR1* (MIM 219100).

Autosomal recessive cutis laxa type IIA (ARCL2A; MIM 219200) is the most common type of CL and is characterized by variable clinical features including skin abnormalities, growth and developmental delay, and skeletal, neuromuscular, and central nervous system involvement [2]. ARCL2A also shares clinical presentations with wrinkly skin syndrome (WSS; MIM 278250), which appears to be a milder form of ARCL2A. Biallelic *ATP6V0A2* mutations are responsible for both ARCL2A and WSS [3].

Loss-of-function mutations in *ATP6V0A2* eventually lead to premature intracellular aggregation of tropoelastin, which in turn prevents mature elastin deposition in the extracellular matrix. Hucthagowder et al. [4] showed that abnormal elastin biosynthesis is the key pathophysiological mechanism affecting skin and connective tissue but does not underlie the neurological and developmental findings in ARCL2A. Impaired secretion of other proteins that have functions in nervous and skeletal tissues may give rise to additional clinical manifestations [4]. In ARCL2A, other mutations affecting assembly and functioning of the complex cause phenotypes overlapping with ARCL2A [5].

We present two siblings with some manifestations of ARCL2A plus unusual findings of bleeding diathesis, defective wound healing, and bilateral ectopia lentis. We identified a causative mutation and performed microscopic studies to investigate the cellular pathology.

## Materials and Methods

### Patients

**Figure 1:**
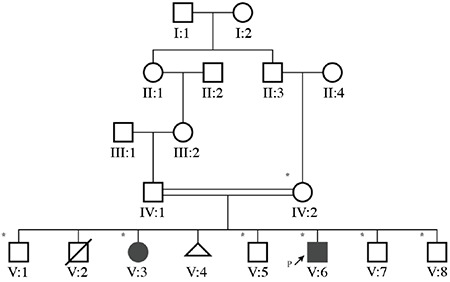
Pedigree of the family. Individuals included in the genetic study are indicated with asterisks. Patients diagnosed with cutis laxa are depicted in black. P: Proband.

The consanguineous parents (Figure 1) have six children, and the daughter and a son (V:3 and V:6, respectively) have some manifestations of ARCL2A. The blood group of both patients is A Rh-positive. Another son (V:1) has intellectual disability (ID) only. A son had died of cardiac anomaly at the age of two months, and a miscarriage at 20 weeks was also reported.

## Methods

### Genetic Analysis

Single nucleotide polymorphism (SNP) genotyping was performed for the mother and all six siblings using Illumina OmniExpress-24 BeadChip targeting >700,000 SNP markers. Multipoint linkage analysis was performed using the GeneHunter program within the software package EasyLinkage [6,7]. Logarithm of odds (LOD) scores were calculated assuming an autosomal recessive model, full penetrance, and a disease allele frequency of 0.001. Regions of >2 Mb and with shared homozygosity in patients were considered. A DNA sample of patient V:6 was subjected to exome sequence analysis to identify the putative causative mutation. Sequencing data were analyzed according to GATK Best Practices recommendations using human reference genome hg19 [8]. Variants in the linked region that were possibly harmful to the protein were considered. Furthermore, variants in the 39 known inherited platelet disease genes (Table 1) were evaluated for any possible contribution to bleeding diathesis.

### Light Microscopy

Skin samples were obtained from the anterior arm by minor surgery and fixed in 4% paraformaldehyde and 2.5% glutaraldehyde in 0.1 M sodium cacodylate buffer (pH 7.2). After a wash in buffer solution, samples were dehydrated through a graded ethanol series and then embedded in paraffin blocks. Sections of 4 μm in thickness were obtained using a microtome and collected on slides, which were subsequently stained with dyes hematoxylin and eosin, Masson trichrome, and periodic acid-Schiff (PAS).

### Transmission Electron Microscopy

Blood samples were centrifuged at 300 x g for 15 min to collect platelets in the buffy coat. Then both these buffy coat pellets and skin samples sized about 1 mm^3^ were fixed in 4% paraformaldehyde and 2.5% glutaraldehyde in 0.1 M sodium cacodylate buffer (pH 7.2). After washing in buffer solution, samples were postfixed in 1% OsO_4_ in 0.1 M sodium cacodylate buffer (pH 7.2), dehydrated through a graded ethanol series, and embedded in Epon-812 resin. Ultrathin sections were cut with an EM UC6 ultramicrotome (Leica Microsystems), and sections were collected on Formvar-coated copper grids, stained with uranyl acetate, and lead-stained to enhance contrast. Grids were viewed with a JEOL JEM 1010B transmission electron microscope operating at 80 kV, and images were obtained with a Megaview II digital camera and AnalySIS software (Soft Imaging System GmBH, Germany)

## Results

### Clinical Findings

### Patient V:6

The proband is a 21-year-old male who was referred to our hematology clinic for evaluation of bleeding diathesis prior to a left mastoidectomy operation due to chronic suppurative mastoiditis. He had wrinkled skin, hyperpigmentation, microcephaly, dysmorphic facial features, cleft lip and palate, and ectopia lentis. Bleeding diathesis, delayed wound healing, and easy bruising was noticeable in early childhood. He had been operated on for cleft lip and palate at age 6 months and for undescended testis and inguinal hernia at age 10 years. He had a history of chronic suppurative otitis media attacks that eventually caused sensorineural hearing loss. After a mild trauma to the left tibial region at age 20 years, a deep wound developed and progressed to acute compartment syndrome. He was hospitalized, and a fasciotomy was performed. During this period, excessive bleeding requiring blood transfusion attracted attention. Complete blood count showed white blood cells of 4.87x10^3^/µL (N: 4-10x10^3^/µL), hemoglobin of 10.9 g/dL (N: 12-16 g/dL) with mean corpuscular volume of 72 fL (N: 80-94 fL), and platelet count of 205x10^3^/µL (N: 150-400x10^3^/µL) with mean platelet volume of 13.6 fL (N: 9-11 fL). Hypochromic and microcytic red blood cells and large platelets were seen on peripheral blood smear. Ferritin level was low (14 ng/mL, N: 20-150), and hemoglobin electrophoresis was normal. Iron deficiency anemia was treated with oral therapy. Prothrombin time, activated partial thromboplastin time, D-dimer, fibrin degradation products, and fibrinogen activity were found to be normal. Skin bleeding time (Ivy method) was 16 min (N: 4-9 min), and PFA-100 revealed prolonged closure times; both collagen/EPI (N: 85-157 s) and collagen/ADP (N: 65-125 s) results were >300 s. Light transmission aggregometry studies showed slightly decreased aggregation with ADP, collagen, and epinephrine. Aggregation with ristocetin was normal. Flow cytometric analysis of the peripheral blood platelets revealed normal expression with CD41 (for glycoprotein IIb), CD61 (for glycoprotein IIIa), CD42a (for glycoprotein IX), and CD42b (for glycoprotein Ib) cell surface markers. von Willebrand factor (*vWF*) activity to antigen ratio was low (0.35, N: >0.7), and factor VIII activity was 74% (N: 50%-150%).

### Patient V:3

The elder patient is 35 years old. She had wrinkled skin, hyperpigmentation, microcephaly, dysmorphic facial features, cleft lip and palate, ectopia lentis, hearing loss due to chronic suppurative otitis media, bleeding diathesis (menometrorrhagia requiring oral and parenteral iron treatment), easy bruising, and defective wound healing. She had been operated on for cleft lip and palate, and has had eye operations for ectopia lentis and bilateral corneal transplantations for corneal clouding. Papillomatous lesions were noticed on her tongue. Complete blood count revealed hypochromic and microcytic anemia consistent with iron deficiency, normal white blood cell count, and differential and normal platelet count with slightly elevated mean platelet volume. Basic coagulation tests (prothrombin time, activated partial thromboplastin time, D-dimer, fibrin degradation products, and fibrinogen activity) were normal. Skin bleeding time (Ivy method) was 15 min (N: 4-9 min). Light transmission aggregometry studies showed normal aggregation with ADP, collagen, ristocetin, and epinephrine. *vWF* activity to antigen ratio was low (0.3, N: >0.7), and factor VIII activity was 70% (N: 50%-150%).

**Figure 2:**
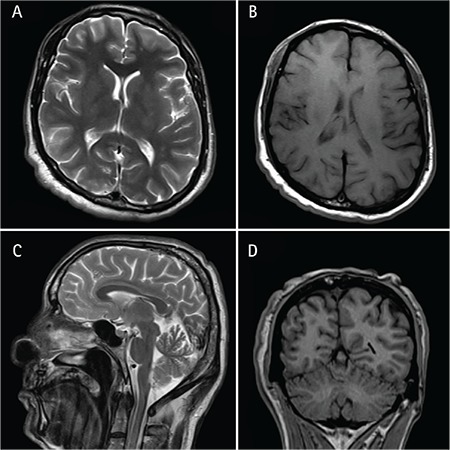
Magnetic resonance imaging of patient V:6 in axial (A and B), sagittal (C), and coronal (D) planes.

Neurological examination and cranial magnetic resonance imaging (MRI) of both patients were normal, excluding structural defects besides microcephaly (Figure 2). We concluded that the disease was atypical cutis laxa.

### Genetic Findings

Initial Sanger sequencing of platelet surface glycoprotein genes *GP1BA, GP1BB, GP9, ITGA2B*, and *ITGB3* did not reveal any mutations in the patients. We thus performed multipoint linkage analysis to localize the disease gene. We obtained a single region of >2 Mb, with a LOD score of 2.59. The identified disease locus was 13.7 Mb at 12q24.21-24.32. The region harbored 198 genes, of which 112 were protein coding, including *ATP6V0A2*.

**Figure 3:**
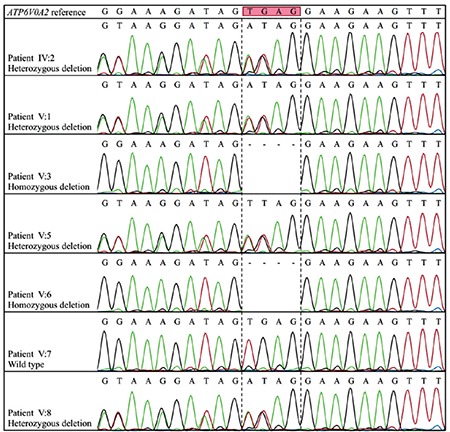
Chromatograms showing novel 4-bp deletion c.2085_2088del (p.(Ser695Argfs*12)) in ATP6V0A2. Sequencing was performed using the reverse primer. Deletion site is marked with a dashed line.

Filtering exome variants in the region resulted in four rare variants (minor allele frequency of <0.01) located in either exons or splice sites (Table 2). A four-base pair frameshift deletion (NM_012463.3, c.2085_2088del, p.(Ser695Argfs*12)) in *ATP6V0A2* exon 17 was the strongest candidate since the gene is known to be responsible for ARCL2A. The variant is deduced to create a premature stop codon leading to a truncated protein of 694 native and 12 non-native amino acids instead of the wild-type 856. The variant is classified as pathogenic according to the American College of Medical Genetics and Genomics guidelines [9]. Sanger sequencing revealed that the variant segregated with the disease in the family (Figure 3).

No pathogenic variant in the known inherited platelet disease genes including *VWF* was detected in Patient V:6. The two patients and a healthy sibling (V:5) shared a heterozygous genotype around the *VWF* gene region. Thus, we ruled out involvement of any *VWF* defect in the bleeding diathesis and defective wound healing findings in the family.

### Microscopy Findings

**Figure 4:**
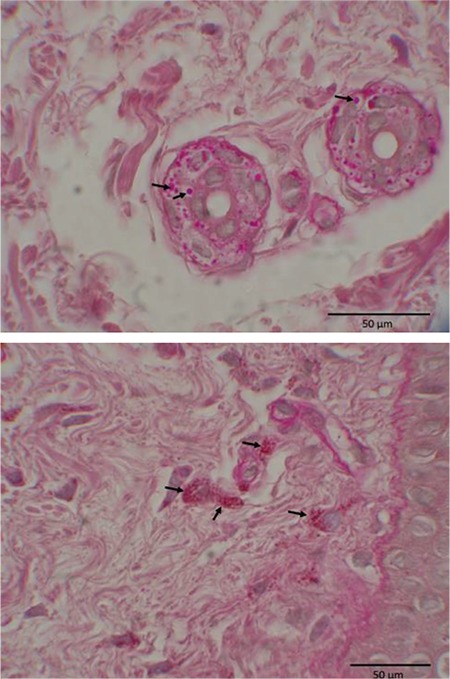
Light microscopic pictures of dermis and eccrine sweat glands with intracellular periodic acid-Schiff positive inclusions in patient V:3.

**Figure 5:**
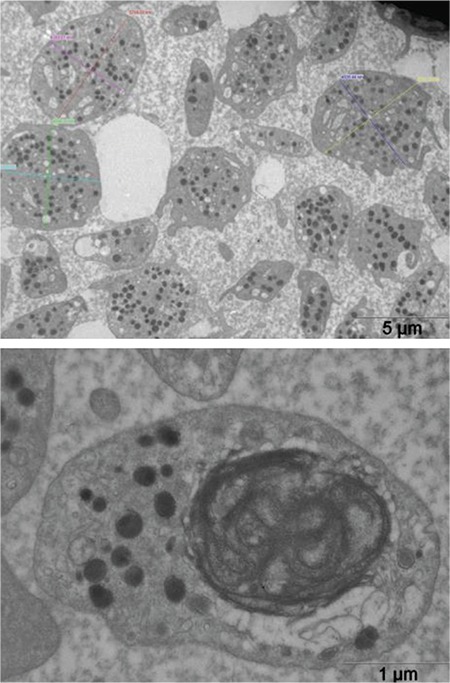
Electron micrograph of the granules resembling polyglucosan content similar to Lafora bodies in dermal fibroblasts of patient V:3.

We studied platelets and dermal tissues under a transmission electron microscope as well as a light microscope to investigate the underlying cause of bleeding diathesis. The most prominent finding was the presence of PAS-positive intracytoplasmic granules in a number of cells in dermal and epidermal tissues of both patients (Figure 4). Under the electron microscope, the content of those granules was assessed to be similar to polyglucosan bodies, and some of them resembled those seen in Lafora disease, i.e. Lafora bodies (Figure 5). They were variable in size and contained electron-dense and lipid-like inclusions in fibroblasts, exocrine glandular epithelial cells, and epidermal basal cells.

**Figure 6:**
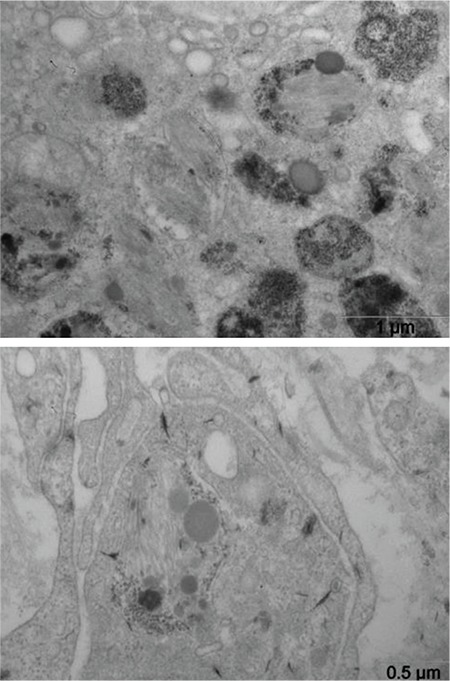
Electron micrographs showing large platelets and rare lamellar inclusions in platelets from patient V:3.

In both patients a decreased quantity of elastic fibers in dermis and subcutis layers was observed under the light microscope. Electron microscopic evaluation of platelets for bleeding diathesis did not show any prominent abnormality except for the presence of some large platelets (approximately 5 µm in diameter; N: 2-4 µm) in both patients. In addition, very rare lamellar inclusions were found in platelets of patient V:3 (Figure 6).

## Discussion

ARCL2A is a clinically highly variable group of connective tissue disorders characterized by inelastic skin due to lack of mature elastin fibers in the extracellular matrix. Highly heterogeneous clinical findings with respect to organ involvement and severity have been reported [10,11]. Loss-of-function mutations in various subunits of the V-ATPase complex cause similar diseases resulting from impaired glycosyltransferase activity and organelle trafficking [5].

We identified a novel frameshift deletion in *ATP6V0A2* that is deduced to result in the truncation of the protein that would cause loss-of-function for the V-ATPase complex. CL patients with biallelic *ATP6V0A2* mutations generally suffer from additional findings such as growth and developmental delay as well as neurological and skeletal anomalies. Our patients do not have growth or developmental delay; their heights are 165 and 175 cm (V:3 and V:6, respectively) and secondary sexual features are fully developed. Eye features in ARCL2A are downslanting palpebral fissures, strabismus, and myopia (MIM 219200). Of those, our patients had only downslanting palpebral fissures but there was additionally ectopia lentis in both and corneal clouding in V:6, features that have not been reported as associated with the disease. Neurologic examination and cranial MRI were normal for both patients, excluding any structural defect besides microcephaly.

The only neurological sign in our patients is ID. As another sibling also has ID, we investigated whether the trait could be due to a common mutation in the three siblings. We performed linkage analysis assuming all three as affected to map the putative trait locus but did not find a candidate locus. We concluded that this common trait possibly does not have the same etiology in the three siblings. Any contribution of the KDM2B variants (Table 2) in the development of ID in patients V:3 and V:6, a characteristic finding of ARCL2A, remains elusive.

ID, facial dysmorphism, and microcephaly have been reported in ARCL2A [2], but not bleeding diathesis or defective wound healing. Those latter two phenotypes could be a unique manifestation of the novel *ATP6V0A2* mutation in the family or due to a mutation in another gene, but we did not detect a candidate mutation that could possibly underlie the trait. Since platelet functions and levels of coagulation factors were normal in both patients, we investigated skin biopsy samples and platelets from peripheral blood by electron microscopy. Ultrastructural studies did not reveal any abnormal features in the fine structure of the platelets except for larger platelet sizes. Patient V:6 did not carry any pathogenic variant of known inherited platelet disease genes; therefore, the cause of the larger size of the platelets remains elusive. In skin tissues decreased elastin fibers were observed, which is hypothesized to underlie the wrinkled skin. Furthermore, we noticed intracytoplasmic inclusions mainly in dermal fibroblasts, exocrine glandular epithelial cells, and epidermal basal cells, but these were present also in the unaffected members of the family. The content of those inclusions remains unknown.

Bleeding diathesis, easy bruising, and defective wound healing in our patients could be the consequence of the abnormal elasticity of the extracellular matrix as in the case of Ehlers-Danlos syndrome, in which affected skin elasticity leads to bleeding tendency. Although there was a slight decrease in platelet aggregation in patient V:6, the expression of platelet surface glycoproteins was normal. In addition, *vWF* activity to antigen ratios were low in our patients. A low *vWF* activity to antigen ratio is a typical sign of *vWF* disease type 2A (MIM 613554), which is caused by *VWF* gene defects. Since we excluded a possible contribution of *VWF* defects, we hypothesized that possible abnormalities in posttranslational modifications of *vWF* could have contributed to bleeding diathesis and defective wound healing, similar to the situation in *vWF* disease type 2A. *vWF* is subject to extensive posttranslational modifications such as sulfation, glycosylation, multimerization, and proteolytic cleavage to create functional *vWF*, which is required for proper platelet adhesion [12,13,14]. Wagner et al. [15] concluded that some multimerization of *vWF* occurs in the Golgi apparatus, and a proton pump defect can lead to incomplete multimerization as in von Willebrand disease type 2A. Alternatively, bleeding diathesis in our patients could be related to possible impaired glycosylation of platelet surface glycoproteins, as O-glycosylation of platelet surface glycoproteins is important for their functions [16]. Whether glycosylation of platelet surface glycoproteins is impaired in ARCL2A and whether these two traits (bleeding diathesis and defective wound healing) are part of ARCL2A remains to be elucidated in future studies, as it is likely that not all reported ARCL2A cases have been subjected to a thorough hematological investigation.

## Conclusion

We have identified a novel mutation in *ATP6V0A2* causing autosomal recessive CL. Our patients presented with multisystemic CL phenotypes but strabismus, myopia, and growth or developmental delay were not observed. Brain malformations besides microcephaly were not present and additional clinical findings of bleeding diathesis, defective wound healing, ectopia lentis, and corneal clouding have not been reported previously; hence, our findings expand the clinical and allelic spectrum of the disease. We suggest that ARCL2A patients could benefit from detailed hematological examinations, especially prior to surgical operations.

## Figures and Tables

**Table 1:**
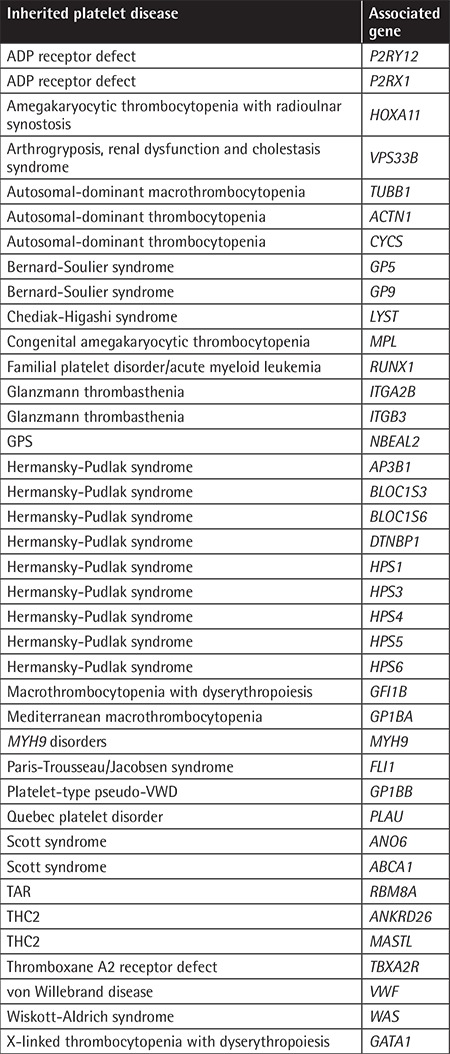
Inherited platelet disease-related genes.

**Table 2:**
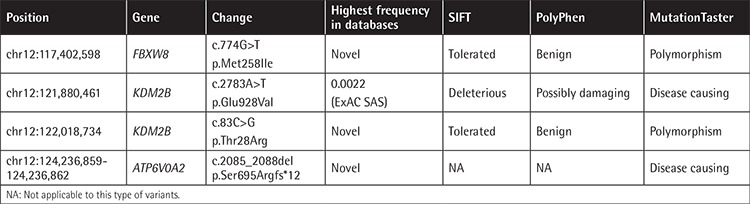
Homozygous rare/novel variants affecting amino acid sequence at the identified disease locus.
